# Inflammatory myofibroblastic tumour: unravelling the interplay of inflammation, kinase signalling, and therapeutic resistance

**DOI:** 10.3389/fonc.2026.1891320

**Published:** 2026-07-21

**Authors:** Sara Maiti, Archana Rose Reji, Swathi Senthil, Rakshana R., Rekha Solairaju, Rehana Kunjumon, Payel Ghosh, Syama H.P., Pritam Sadhukhan, Arkajyoti Dutta

**Affiliations:** 1 School of Bio Sciences and Technology, Vellore Institute of Technology, Vellore, Tamil Nadu, India; 2 Department of Cellular, Molecular and Genetic Medicine, Virginia Commonwealth University, Richmond, VA, United States

**Keywords:** alectinib, ALK inhibitors, anaplastic lymphoma kinase (ALK), epithelioid inflammatory myofibroblastic sarcoma (EIMS), gene fusion, immune microenvironment, inflammatory myofibroblastic tumour (IMT)

## Abstract

Inflammatory myofibroblastic tumour (IMT) is a mesenchymal tumour characterised by myofibroblastic cells and the presence of inflammatory cells, such as macrophages, neutrophils, and lymphocytes. IMT can originate from diverse anatomical locations within the body. The macrophage-rich immune microenvironment contributes to cytokine release and inflammation, but IMT is primarily driven by oncogenic alterations. Oncogenic fusions, most commonly involving anaplastic lymphoma kinase (ALK), and other genes, such as ROS1 and NTRK3, are seen in most IMTs. Upregulation of these genes activates downstream signalling pathways such as RAS/MAPK and PI3K/AKT, leading to uncontrolled cell proliferation. Constitutive tyrosine kinase activation disrupts cell-cycle control by suppressing p21/p27, inactivating Rb, and impairing p53-dependent checkpoints, thereby limiting apoptosis. Combining surgery with targeted kinase inhibitors remains the primary therapeutic approach, especially for ALK-positive IMT. On the contrary, ALK-negative IMT harbours distinct oncogenic fusions and is driven by diverse signalling pathways. Thus, it requires therapeutic methods targeting the specific molecular alterations. Resistance to ALK inhibitors can emerge during treatment. This resistance arises through bypass signalling, epigenetic changes, and secondary mutations. This review aims to analyse the interplay between inflammation and genetic fusions in the origin and progression of IMT. The review provides an overview of the therapeutic approaches currently in use, mechanisms of treatment resistance, and potential techniques to improve diagnosis and develop personalised therapeutic strategies.

## Graphical Abstract

**Figure:**
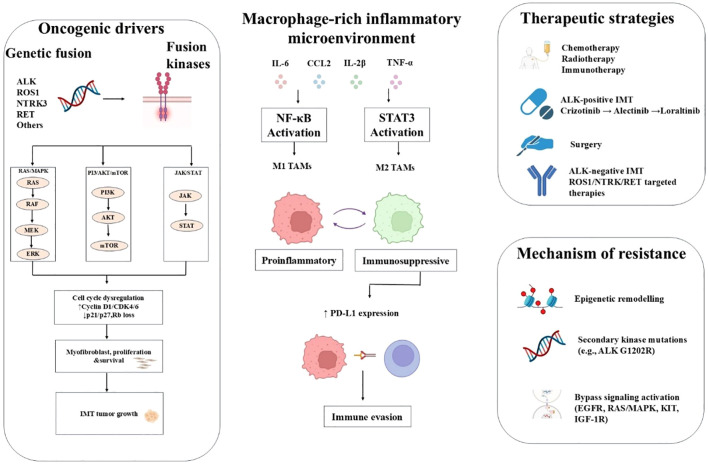

## 1 Introduction

IMT is a rare malignant tumour of mesenchymal origin that occurs in tissues such as bone, cartilage, muscle, and connective tissue (1). It is characterised by a mixture of myofibroblastic spindle cells and inflammatory cells, including lymphocytes, plasma cells, and eosinophils (2). IMT is initiated by oncogenes via gene fusions and appears to be a histological lesion caused by inflammation (3). In 1999, it was noted that IMT was similar to Anaplastic Large Cell Lymphoma (ALCL) in that ALK exhibited gene rearrangements leading to ALK fusion genes and continuous proliferation and tumour formation. In 2006, the World Health Organisation classified IMT as a locally aggressive neoplasm because it has low-grade malignant potential and is neither fully benign nor fully malignant (1). IMT is reported to affect 1 in 1,000,000 individuals in a population with approximately 150–200 new cases diagnosed annually in the United States (4). Based on location, IMT shows varied sites of occurrence, with the liver and biliary tract being the most common, accounting for up to 31.8% of reported cases, followed by the head and neck regions (20.6%) and the lungs (18.2%), and the urogenital system (7.4%) (5).

Inflammatory component is the basic criterion for diagnosis in an IMT, but the quantity and nature of inflammatory cells can differ between cases, making the diagnosis more difficult (6). Laboratory tests reveal an inflammatory response, including elevated white blood cell counts, elevated levels of inflammatory markers such as C-reactive protein, and a high erythrocyte sedimentation rate. High immunoglobulin levels, increased blood platelet count and other markers of long-lasting inflammation are found as they persist throughout the disease (7).

Genetic analysis using advanced techniques can identify that IMT is not solely due to inflammation but is primarily driven by oncogenes. The most important genetic feature of IMT reported in almost 80% cases is the presence of a mechanism called kinase gene fusion, where abnormal fusion of two separate genes occurs that leads to uncontrolled cell growth and division (8). These genetic changes mostly occur in the gene encoding Anaplastic Lymphoma Kinase (ALK), located on chromosome 2p23, which is involved in genetic fusions and encodes a tyrosine kinase that regulates cell growth. This fusion keeps ALK constitutively active, driving uncontrolled cell growth and tumour formation (9, 10).

## 2 Epithelioid inflammatory myofibroblastic sarcoma: the aggressive variant

The aggressive nature of EIMS could be due to the fusion gene partners involved. The occurrence of *RANBP2- ALK* fusions is common in this subtype. Fusion between exon 18 of RANBP2 and exon 20 of ALK results in constant ALK activation, uncontrolled cell growth, and increased tumour invasiveness. Other ALK fusion partners reported to have aggressive clinical behaviour in EIMS are *RRBP1-ALK* and *EML4-ALK* fusions (11, 12). RANBP2 on chromosome 2q13 was positively associated with better IMT/EIMS differentiation. This is the most frequent fusion partner in EIMS cases. RRBP1-ALK is another fusion partner seen in EIMS and is associated with poor prognosis and rapid disease progression. Among the 58 reported cases, 38 occurred in males, indicating a male predominance that does not appear to affect disease severity (13, 14). It is alarming that the early mortality rate in EIMS is higher. Approximately 22 patients died within the first year of diagnosis, reflecting the rapid progression, aggressive nature, and poor prognosis of the disease (15).

EIMS shows distinct microscopic and molecular features, with cells being round or epithelioid in shape. The ALK staining in EIMS shows a perinuclear membrane pattern, indicating an aggressive phenotype. EIMS grows rapidly and recurs more quickly than the classic IMT. Hence, in 2011, it was classified separately from IMT (16). To date, around 60 cases have been reported with EIMS, although the actual number is expected to be higher due to underdiagnosis. The aggressive nature of EIMS could be due to the fusion gene partners involved. The occurrence of *RANBP2- ALK* fusions is common in this subtype. Fusion between exon 18 of RANBP2 and exon 20 of ALK results in constant ALK activation, uncontrolled cell growth, and increased tumour invasiveness. Other reported ALK fusion partners with aggressive clinical behaviour in EIMS include *RRBP1-ALK, EML4-ALK*, and *VCL-ALK* fusions (14).

Among the 58 reported cases, as shown in **Table 1**, 38 are male, suggesting a male predominance, but this does not influence disease severity (37). The higher rate of early mortality in EIMS is an alarming concern. Around 22 patients died within one year of diagnosis, suggesting its rapid progression, aggressive nature and poor prognosis. Accurate diagnosis also requires careful exclusion of conditions that closely resemble IMT, like IgG4- related disease, inflammatory pseudo-tumour, fibrosarcoma, and other spindle cell tumours (38).

**Table 1:** Biochemical characters and treatment methods used in IMT: includes data from 17 case reports and 4 studies.

SL. no.	Region of occurrence	No. of patients	ALK status	Mode of treatment	Survival status	Reference
1.	Intercranial	1	+ NPM1-ALK fusion	Complete Surgical Resection	Survived	(17)
2.	Intercranial	1	+ SQSTM1-ALK	Complete Surgical Resection	Survived	(17)
3.	Lung	1	–	Surgery	Survived	(18)
4.	Lung	1	+	Alectinib, later Lorlatinib	Survived	(19)
5.	Lung	1	+	Alectinib, later Chemotherapy	Survived	(20)
6.	Abdominal	1	+	Surgery, Crizotinib, later Alectinib	Deceased	(21)
7.	Submandibular	1	+	Surgery	Survived	(22)
8.	Urachal	1	+ FN1-ALK fusion	Surgery, laparoscopic resection	Survived	(23)
9.	Lung	1	+ PLEC-EML4-ALK fusion	Surgery, AKI inhibitors, Radiation	Deceased	(24)
10.	Lung	1	NA	Surgery	Survived	(25)
11.	Lung	1	–	Surgery	Survived	(26)
12.	Urinary Bladder	1	+	Surgery	Survived	(27)
13.	Intracranial	1	NA	Surgery, radiotherapy Immunotherapy	Survived	(28)
14.	Lung	1	–	NSAID	Survived	(29)
15.	Lung	7	+ ALK fusion- 4	Surgery	Survived	(30)
16.	Lung	1	+ EML4 E7-ALK fusion	Surgery, later Alectinib	Deceased	(30)
17.	Lung	1	–	Surgery	Deceased (12 years later)	(31)
18.	Lung	1	–	Surgery Chemoradiotherapy	Survived	(32)
19	Skull Base	1	–	Corticosteroids later Radiotherapy	Survived	(33)
20	Trachea	1	+	Surgery, Radiotherapy	Survived	(34)
21	Head and neck	45	NA	Surgery, 20 patients- surgery + radiotherapy	32 Survived 13 Deceased	(34)
22	Diverse (Pediatric)	16	+ 10	Surgery, 1 ALK inhibitor therapy	Survived	(35)
23	Uterine	27	+ 16 - 2 NA 9	Surgery 1Surgery+Chemotherapy	Survived	(35)
24	Diverse	72	+ 30 - 36 Unknown 6	Surgery Therapy after recurrence	6 Deceased	(36)

Terms: Urachal – a fetal structure that connects the bladder to the umbilical cord. Laparoscopic – using a laparoscope, a thin tube with a camera, to view internal organs.

## 3 Chronic inflammation and tumorigenesis

Cytokines such as tumour necrosis factor α (TNF-α), interleukin-6 (IL-6), and interleukin-1β (IL-1β) activate signalling proteins, including NF-κB and STAT3. These cell signalling proteins activate genes that promote the survival and growth of cells, and also the formation of new blood vessels (39, 40). In IMT, these same pathways are also activated by gene fusions, mostly involving *ALK* or *ROS1*. This suggests that genetically driven malignant growth is closely linked to inflammatory signalling. Continuous activation of NF-κB and STAT3 prevents normal cell death, promotes myofibroblastic overgrowth, and maintains an inflammatory tumour microenvironment (18).

### 3.1 Immune microenvironment signature

Inflammatory Myofibroblastic Tumour (IMT) has been found to have a unique balance between inflammation and tumour-promoting factors in the immune microenvironment. The tumour microenvironment is rich in macrophages, lymphocytes, and plasma cells. This immune-rich environment influences tumour behaviour (3). The most commonly seen immune cells in thoracic IMT tissues are macrophages (CD68^+^). Apart from the macrophages, there are moderate numbers of CD4^+^ helper T cells, CD8^+^ cytotoxic T cells, CD20^+^ B cells and some FOXP3^+^ regulatory T cells (30). This visualisation is done using multiplex immunofluorescence profiling of thoracic IMT tissues. A primarily innate immune environment is shown by this distribution. This pattern reflects limited adaptive immune activity and is consistent with the generally slow tumour growth (21).

In the majority of IMTs, the ALK gene is rearranged and, in some cases, NTRK3 expression is elevated. The genetic changes are then accompanied by an expansion of macrophages in the tumour, a sign of active immune signalling. Cytokines and chemokines produced by tumour cells, like IL-6 and TNF-α, recruit and activate macrophages. On the contrary, the mechanisms of immune tolerance are less common in IMTs than in more aggressive tumours due to a relatively lower amount of regulatory T cells (30). Studies of the IMT have found a relationship between this microenvironment, which is rich in macrophages and antigen presentation, and immune activation. Macrophages within IMT clear cellular debris and contribute to tissue remodelling. Through their interaction with T cells, they also influence the balance between inflammation and tissue repair (24).

PD-L1 is continuously expressed on both tumour and tumour-associated macrophages (TAMs) in IMT, and its engagement of PD-1, which is present on cytotoxic T cells, reduces anti-tumour immunity, resulting in lesions persisting despite abundant immune infiltrate (41). TAMs present in IMT are not uniform: M1 polarised macrophages release pro-inflammatory mediators, including TNF-α and IL-2, which support antigen presentation, whereas M2 polarised macrophages secrete IL-10, TGF-β and VEGF, favouring PD-L1 upregulation, angiogenesis, immune evasion and tissue remodelling (42). IL-2 secreted in these microenvironments activates STAT3 in both tumour and stromal cells, leading to the formation of an IL-6/STAT3 loop. Activated STAT3 upregulates IL-6 and chemokine production, promotes macrophage polarisation towards M2 and PD-L1-high phenotypes, and simultaneously drives myofibroblast proliferation and survival through downstream ALK/ROS1 fusion signalling. Altogether, PD-L1 expression and the M1/M2 balance within the IMT microenvironment represent plausible biomarkers for immunotherapy and combination targets, along with ALK-directed therapy (42).

## 4 Mechanisms of action of fusion kinases involving cell-cycle dysregulation

**Figure 1:**
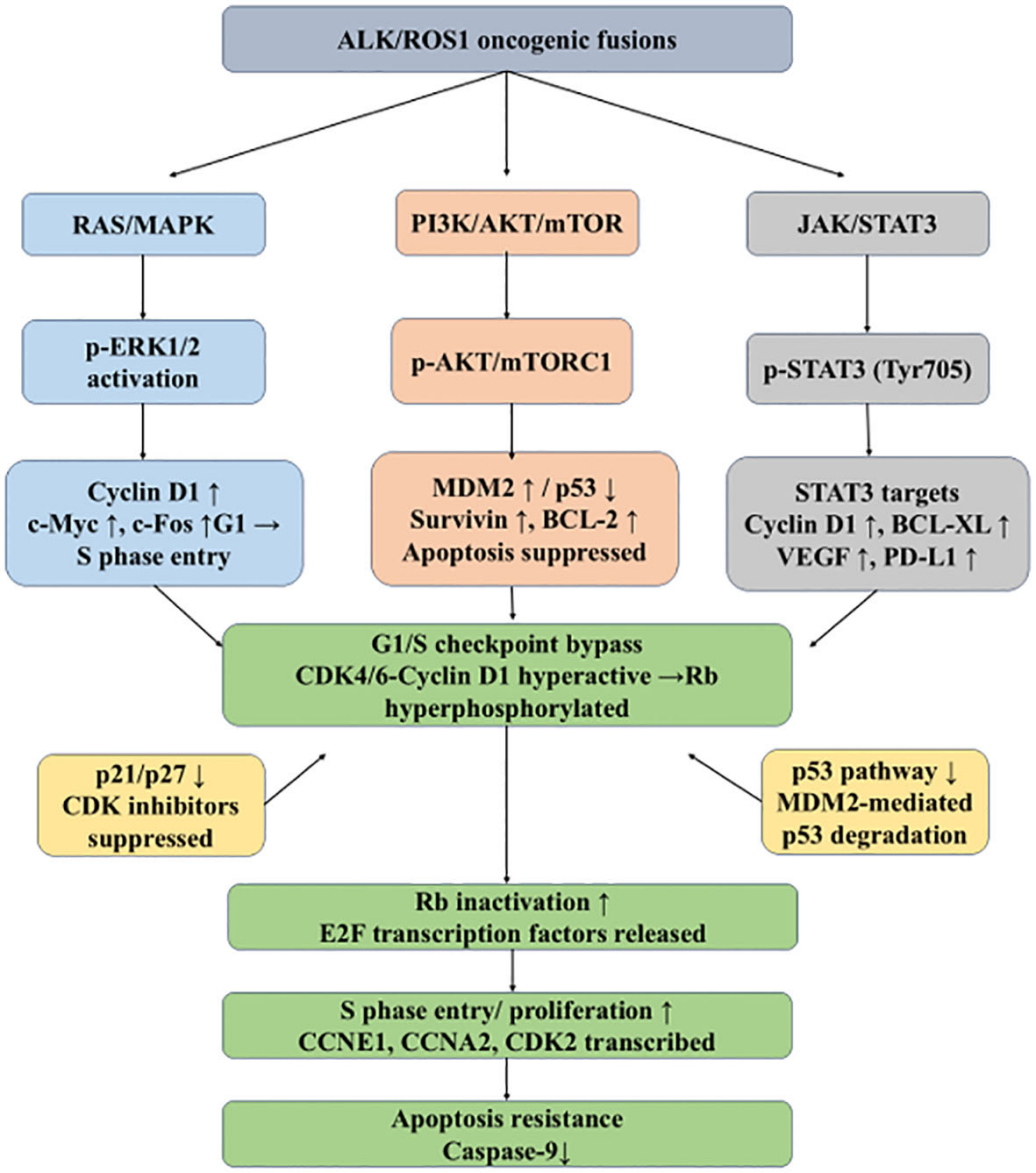
Schematic representation of downstream signalling cascades activated by ALK/ROS1 oncogenic fusion in IMT.

Inflammatory Myofibroblastic Tumour (IMT) frequently has oncogenic fusion kinases (*ALK, ROS1*, and *PDGFRβ*) that stimulate abnormal cell growth. These fusions arise from chromosomal rearrangements that place fusion proteins under constitutive promoter control and activate signalling pathways such as RAS/MAPK, PI3K/AKT, and JAK/STAT (25). In IMT, constitutive receptor tyrosine kinase activity increases cyclin D1 and CDK4/6 levels beyond their normal growth factor–dependent activation. This causes cells to pass through the G1/S checkpoint and reduces apoptosis. The *PI3K/AKT* pathway also protects against cell death and stimulates cell growth by inhibiting proapoptotic molecules and inducing the activity of mTOR signalling (27). The schematic representation of *ALK/ROS1* oncogenic fusion in IMT is illustrated in **Figure 1**.

Some fusion kinases, such as the *RANBP2–ALK* and *TFG–ROS1*, are abnormally localised in cells and disrupt the assembly of the mitotic spindle. This disruption causes chromosomal instability (28, 29). These changes promote tumour development by disrupting cell-cycle control and genomic integrity. Abnormal kinase signalling is suppressed, and normal cell growth is restored with a target inhibitor, such as crizotinib. In particular, remarkable efficacy has been observed in fusion-positive IMTs using these targeted inhibitors (33).

## 5 Comparative biology of ALK-positive and ALK-negative IMT

ALK-positive IMTs are usually driven by well-defined oncogenic mechanisms based on constitutive ALK activation and specific fusion proteins with strong promoters and protein-binding domains. Therefore, ALK-positive IMTs tend to show more uniform clinical behaviour and respond more reliably to ALK-targeted therapies (36, 41). By contrast, ALK-negative IMTs are molecularly heterogeneous. Alternate kinase gene fusions, such as *ROS1, NTRK* or *PDGFRB* fusions, are observed in around two-thirds of ALK-negative tumours. Therefore, without ALK rearrangements, most IMTs are caused by some type of oncogenic activation of these kinases. However, no recognisable kinase fusions are observed in the remaining one-third of ALK-negative IMT cases, and the molecular origins are less clear (10). ALK-positive IMTs typically show a consistent inflammatory infiltrate dominated by plasma cells and lymphocytes, whereas some ALK-negative IMTs, including those that progress to EIMS, show increased neutrophil infiltration and altered tissue architecture (35, 43).

ALK-positive IMTs generally grow more slowly and have a lower risk of distant metastasis, although recurrence can still occur (44). Whereas in ALK-negative cases, unidentifiable kinase drivers lead to more aggressive behaviour with higher recurrence rates and metastasis potential. However, classical ALK-negative IMT cases are similar to the ALK-positive cases, particularly with respect to kinase fusions. This change of paradigm, the oncogene-driven IMT, has made precision medication and targeted therapy a little easier, yielding better response and survival rates for patients (45, 46).

## 6 Therapeutic strategies for IMT – analysing case studies

### 6.1 Surgery

Surgery remains the primary treatment for IMT, with complete tumour removal being strongly preferred over partial resection due to lower recurrence rates. A cohort study reported relapses in around 17% of patients even after surgical removal. Hysterectomy is recommended in uterine IMT cases where fertility is not a concern (47). Duodenopancreatectomy was performed in a 6-month-old child with pancreatic IMT, and the child reported no recurrence. In the cases reviewed, 19 out of 23 patients underwent surgery, highlighting its central role in IMT management (48).

### 6.2 Targeted therapy

**Figure 2:**
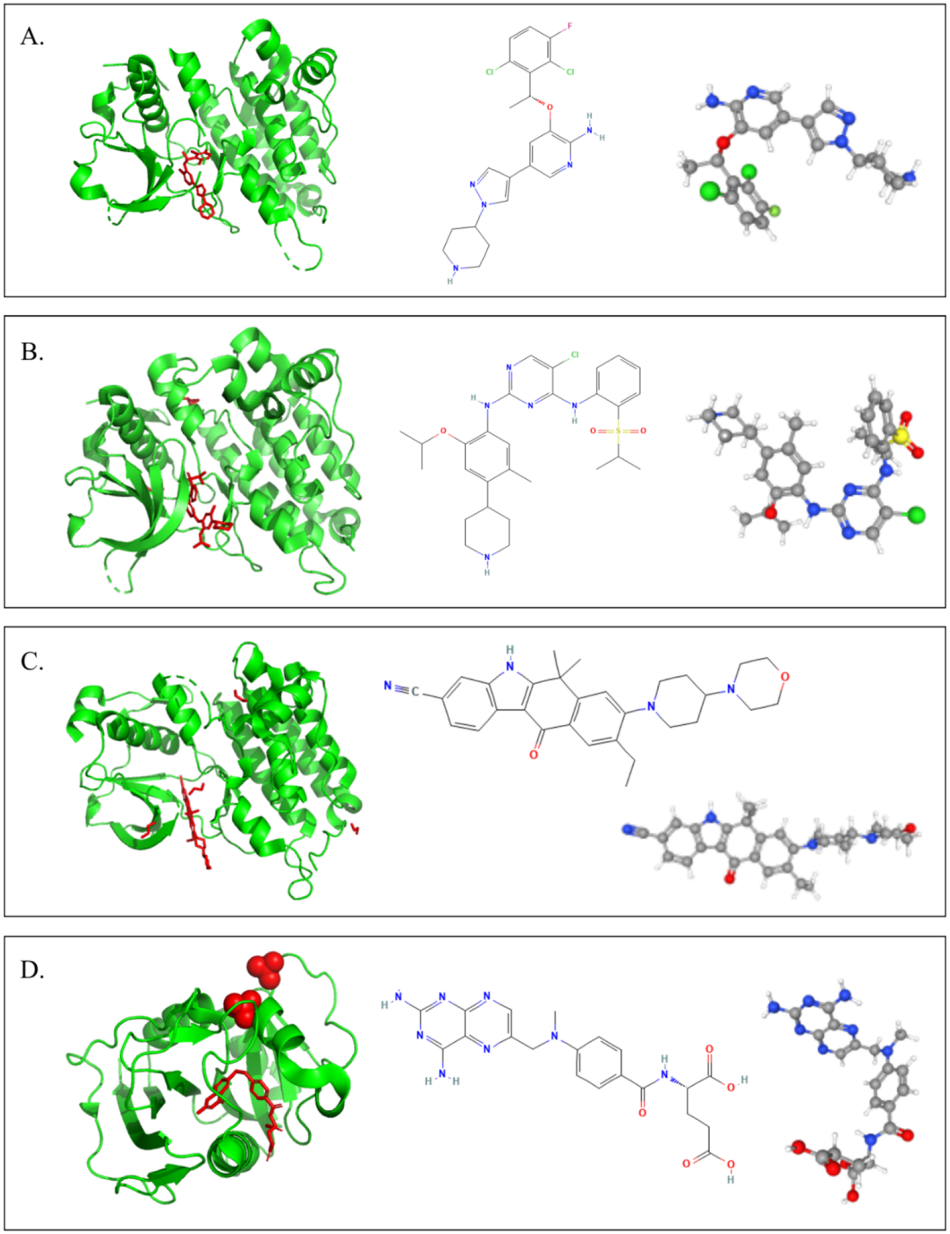
Structure of ALK protein in complex with **(A)** Crizotinib, **(B)** Certinib, **(C)** Alectinib, respectively and **(D)** illustrates the structure of Human Dihydrofolate Reductase complexed with Folate. Methotrexate does not target the ALK protein itself, but rather the folate pathway. The 2D and 3D structures of the drugs are shown alongside.

ALK tyrosine kinase inhibitors (ALK TKIs) are the primary targeted therapy for ALK-positive IMT, including Crizotinib, Alectinib, Ceritinib, and Lorlatinib. Crizotinib, a first-generation ALK TKI, works by binding to the ALK kinase domain, as shown in **Figure 2A**. This binding interaction leads to the blocking of autophosphorylation and downstream signalling pathways (49).

Crizotinib is FDA-approved for ALK-positive IMT that cannot be surgically removed due to size or location. However, resistance to crizotinib has been increasingly reported, often due to ALK amplification or double fusion events that reduce drug occupancy and allow downstream signalling to remain active. When this happens, stronger second-generation ALK inhibitors like Alectinib are used as the next line of treatment (50, 51). A summary of the currently reported targeted therapies and emerging immunotherapeutic agents for ALK-positive IMT is summarised in **Table 2**.

**Table 2:** Summary of targeted therapy and immunotherapy options in ALK-positive IMT.

Treatment name	Molecular target	Study phase	Summary of the main findings	Reference
Targeted therapy				
Crizotinib	ALK	FDA approval supported by multicenter single-arm trials	Strong activity in unresectable, recurrent, or refractory ALK+ IMT; first approved ALK-TKI for ALK+ IMT.	(52)
Ceritinib	ALK	Case reports/case series	Produced partial or complete responses in ALK+ IMT and helped overcome crizotinib resistance in some cases.	(53)
Alectinib	ALK	Case reports/case series	Showed durable responses, including after crizotinib resistance.	(54)
Brigatinib	ALK	Case report; ongoing pediatric/young adult trial	Produced partial response after crizotinib resistance in an ALK-mutated IMT case.	(55)
Lorlatinib	ALK	Case reports/case series	Showed responses in later-line ALK+ IMT, including after resistance to earlier ALK-TKIs.	(56)
Immunotherapy				
Combination immunotherapy with ALK inhibitors	Immune-based therapy plus ALK	Case report	Used as a rescue strategy in refractory metastatic IMT after multiple ALK inhibitor lines.	(57)
Immune checkpoint inhibitors	PD-1/PD-L1 axis	Review-level evidence; scattered case reports	Promising in selected IMTs, especially where immune biomarkers suggest activity, but not established standard therapy.	(28)

**Figure 3:**
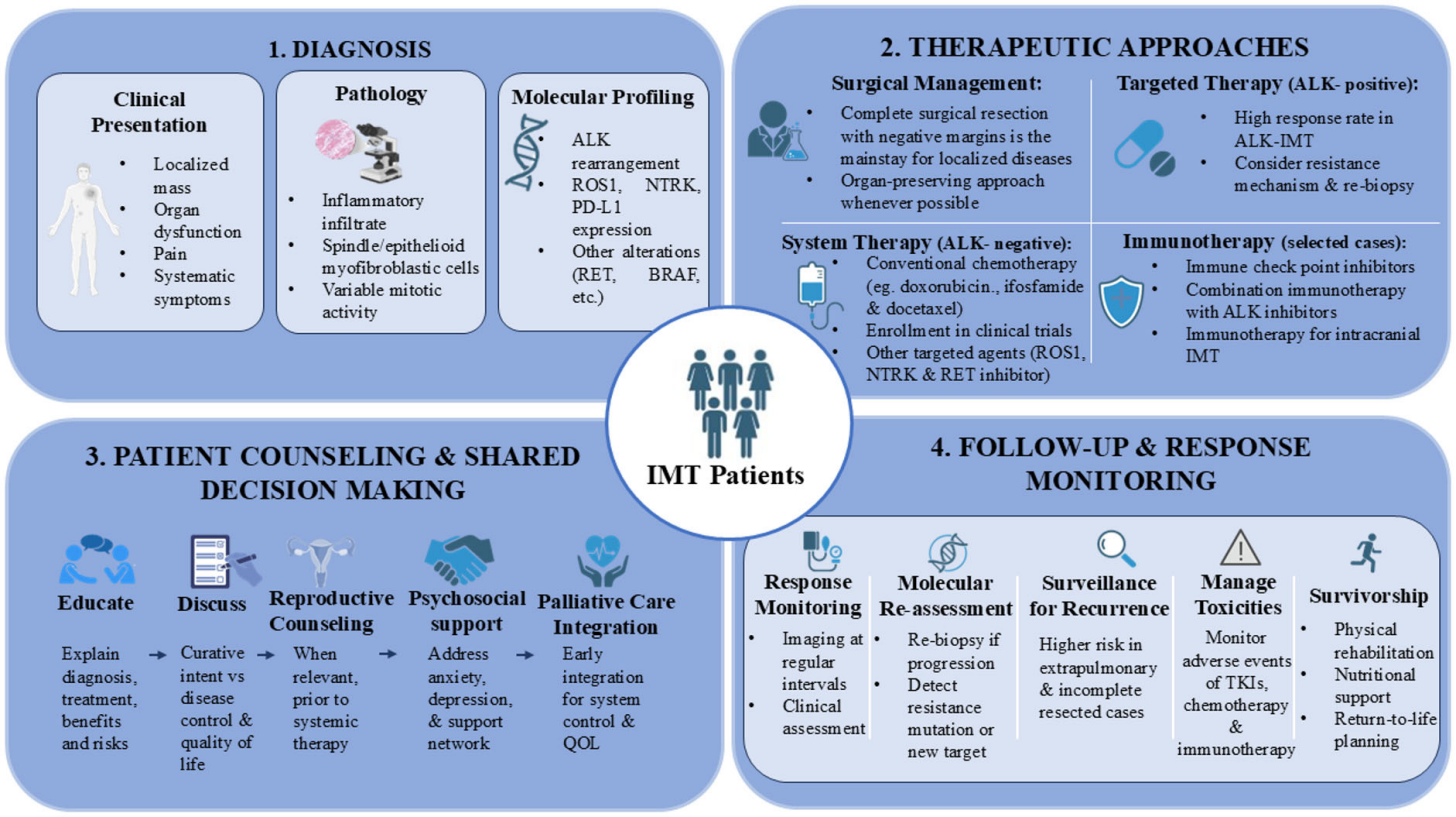
Integrated therapeutic approaches in inflammatory myofibroblastic tumour (IMT).

The ALEX trials (2014–2016), a series of international phase III trials involving 303 ALK-positive NSCLC patients, compared the efficacy of Alectinib and Crizotinib. Patients were randomly assigned to receive either 600mg of Alectinib or 250mg of Crizotinib orally for a median of 17.9 months. Alectinib demonstrated superior outcomes, with a 12-month progression-free survival of 68.4% compared to 48.7% with Crizotinib, and a significantly lower CNS progression rate of 9.4% versus 41.4% (58). It also showed lower toxicity, leading to its FDA approval in 2020 as a first-line treatment for ALK-positive NSCLC and ALK fusion IMT. However, no single ALK inhibitor has yet proven universally effective across all IMT subtypes (59). An overview of the current therapeutic targets for IMT is summarised in **Figure 3**.

It is important to note that the ALEX trial data summarised above are generated from ALK-positive NSCLC, a disease with an immune microenvironment, distinct tumour biology, and a different ALK fusion partner compared with IMT. NSCLC is typically driven by *EML4-ALK* fusion with immune suppressive stroma, whereas IMT is associated with dense macrophage and lymphocyte-rich infiltrate and a broader range of ALK fusion partners (60). To date, no IMT-specific trials comparing alectinib to ALEX have been conducted; the use of ALK inhibitors in IMT is chiefly supported by case reports.

Alongside improvements in the targeted approach, novel immunotherapies are emerging as therapeutic options for specific patients. But so far, immune checkpoint inhibitors have not proven to be a standard therapy for IMT, and there are several reports of their effect in some tumours with programmed death-ligand 1 (PD-L1) expression or other good immunological qualities (61). Further evaluation of IMT in prospective trials is limited, but anecdotal data from heavily pretreated patients suggest that immune checkpoint blockade may be a promising option in ALK-negative tumours or when other targeted therapies are exhausted (62).

Combination strategies combining ALK inhibition with immunotherapeutic agents are also reported. In refractory metastatic patients, sequential ALK-targeted therapy and immunotherapy have been used as rescue therapy following multiple lines of treatment (10). While the available data is confined to anecdotal clinical observations, these experiences highlight the importance of future clinical trials investigating the potential synergistic effects of a combination of targeted and immune therapy (63). The aim of the therapeutic paradigms of IMT and EIMS is to quickly shift from conventional surgery and chemotherapy to biomarker-guided precision medicine. Crizotinib and the growing use of second- and third-generation ALK inhibitors, as well as early investigations into immunotherapeutic strategies, represent important advances over previous reviews. Further multicenter efforts, prospective registries and molecularly stratified clinical trials will be crucial in the development of evidence-based treatment algorithms and outcome enhancement in these rare mesenchymal neoplasms (64).

Resistance to ALK inhibitors remains a significant clinical challenge. A recent study reported a 52-year-old female with ALK-positive uterine sarcoma and lung metastatic IMT who developed Alectinib resistance after 7 months, acquiring a G3604A mutation causing a Glycine-to-Arginine substitution at position 1202 of the ALK gene. She was switched to Lorlatinib, a third-generation ALK inhibitor, but continued to progress. Stability was eventually achieved with epirubicin and cyclophosphamide therapy, with epigenetic modulations suggested as the underlying cause of Lorlatinib resistance (65). A lung IMT with an *ALK-EGFR* fusion that responded well to Alectinib initially but later developed resistance. The patient was switched to Lorlatinib, achieving disease stabilisation without full recovery. RPPA analysis of biopsy samples suggested that EGFR signalling pathways contribute to resistance (65, 66). A case reported that *PLEC-EML4-ALK* double fusion with Crizotinib resistance, where switching to Alectinib led to brain metastases. Lorlatinib combined with radiation was initiated but discontinued due to worsening condition. Additionally, a study reveals that an abdominal IMT that failed to respond to both Crizotinib and Alectinib following surgery (67).

These cases collectively highlight that resistance to ALK inhibitors is not only common but also driven by diverse mechanisms, including secondary mutations, bypass signalling pathways such as *RAS/MAPK*, and epithelial-to-mesenchymal transition, underscoring the dynamic and complex nature of IMT (68). The binding interactions of Crizotinib, Certinib and Alectinib are shown in **Figures 2A-C**, respectively.

### 6.3 Chemotherapy in ALK negative scenario

While surgery remains the primary treatment, chemotherapy serves as an important adjunct in both ALK-positive and ALK-negative IMT. A multicenter European analysis identified anthracycline-based agents and Methotrexate in combination with Vinblastine or Vinorelbine as effective options (68). Other agents such as etoposide, ifosfamide, and cisplatin-containing regimens have also been used in combination, though their efficacy is largely based on small case reports and retrospective studies rather than large clinical trials (69).

In one reported case, an ALK-negative abdominal IMT patient was treated post-surgery with methotrexate, cisplatin, and diclofenac sodium, showing no recurrence on follow-up. For ALK-negative IMT, targeted therapy remains largely unexplored, though ROS1, NTRK, and RET are considered potential targets for future treatment strategies (20).

### 6.4 Radiation therapy

Radiation therapy serves as a useful adjuvant in IMT management. A recent study reported no recurrence or toxicity following total surgical removal of tracheal IMT combined with radiation. In a study of 45 head and neck IMT patients, post-operative radiotherapy did not benefit all patients but significantly improved overall survival in those with malignant transformation. When combined with chemotherapy, radiation has shown promising results. A long IMT case treated with chemoradiotherapy reported progression-free survival exceeding 5 years. The understanding of the tumour’s biological makeup is key to designing an effective chemo-radiotherapy regimen (20). Radiotherapy is also considered when surgery poses significant risks. This study reported a 38-year-old male with skull base IMT deemed inoperable due to its location. Following failed corticosteroid treatment, Fractionated Conformal Radiotherapy (20Gy over 12 days) achieved a complete clinical response, with full recovery after two years (70).

However, radiation therapy is not universally effective, as seen in the case of aggressive metastatic IMT with *PLEC-EML4-ALK* double fusion, where combined Lorlatinib and radiation failed to produce results. The optimal radiation dose remains undefined and varies by tumour location. Overall, radiation therapy holds promise in IMT treatment, but further research into dosage, potency, and combination strategies is needed to refine its role (71, 72).

### 6.5 Immunotherapy

Given the inflammatory nature of IMT, immunotherapeutic agents have emerged as a promising treatment avenue. Sintilimab, a monoclonal antibody that blocks the PD-1 receptor on T cells, prevents cancer cells from evading immune detection by inhibiting the PD-1/PD-L1 interaction. Persistent PD-L1 expression has been reported in IMT (31), supporting its therapeutic relevance. In 2022, 12 cycles of Sintilimab administered to an intracranial IMT patient resulted in the near-complete disappearance of disease signs in approximately 98% of cases (73).

Beyond PD-1 inhibitors, Ghani et al. reported no recurrence in a lung IMT patient following 12 months of Celecoxib, an NSAID, suggesting a potential role for anti-inflammatory agents in IMT management. Camrelizumab, another PD-1 inhibitor, has shown effectiveness in non-squamous NSCLC when combined with chemotherapy, though its application in IMT remains unexplored (74).

## 7 Challenges in IMT

### 7.1 Difficulties in diagnosis

Although computed tomography, ultrasound, endoscopy, and MRI are used to detect and image IMT, diagnosis based on imaging alone is difficult because specific features are lacking. Delayed diagnosis occurs due to the low incidence of IMT, patients with symptom-compatible and other oncogenic tumours (75).

Another reason for the delayed diagnosis of IMT is that there are no recognisable diagnostic criteria, and extensive histological analysis is necessary. The histology analysis, however, is not sufficient to provide an overview of the future disease condition. Retrospective studies also suggest that, in the case of ALK inhibitors, these molecules were effective in IMT therapies, but displayed greater positive responses in patients with non-small cell lung cancer (NSCLC) than in IMT (76).

### 7.2 Immunohistochemical markers

Immunohistochemistry (IHC) is used in the lab to identify specific antigens in a specimen using antibodies conjugated to dyes. Immunohistochemical markers that are important to diagnose IMT include smooth muscle actin (SMA) and anaplastic lymphoma kinase (ALK) (77). SMA shows diffuse expression, whereas ALK is positive only in approximately 50-70% IMT. Partial positivity can be observed with other immunohistochemical markers, such as desmin and muscle-specific actin. Although immunohistochemistry is an important tool to study the physical nature of the tumour, the genetic landscape might not be accurately reflected through IHC (78).

## 8 Future directions: unresolved questions and translational opportunities

### 8.1 Mechanisms of resistance against targeted kinase inhibitors

Kinase inhibition has transformed IMT treatment, yet significant challenges remain, particularly around resistance mechanisms. A key gap lies in understanding how fusion variants such as ALK, ROS1, and PDGFRB influence therapeutic responses, as their structural configurations may affect kinase localisation, dimerisation, and downstream signalling. Detailed biochemical and structural mapping of these fusion proteins could help identify vulnerabilities and guide the design of more effective next-generation inhibitors (79).

Combining molecular diagnostics with flexible treatment algorithms offers a promising path forward. Next-generation sequencing (NGS) can distinguish between on-target mutations, where structural changes in the kinase prevent drug binding, and bypass activation, where parallel signalling pathways are triggered by tumour formation. This distinction enables more dynamic and informed treatment decisions (80). Functional assays such as patient-derived organoids and ex vivo kinase activity profiling can complement genomic data to predict drug sensitivity in real time. Additionally, combining TKIs with small molecules targeting compensatory pathways like MEK, PI3K, or mTOR may help delay the onset of resistance.

Given the rarity of IMT, global collaboration is essential. Centralised registers compiling genomic, clinical, and therapeutic response data across institutions would accelerate biomarker discovery and support the development of evidence-based kinase inhibitor sequencing strategies (81).

Resistance to ALK inhibitors in IMT falls into three broad overlapping categories: bypass signalling, on-target secondary mutations, and non-genetic adaptation. Bypass signalling is the activation of parallel receptor tyrosine kinases or downstream effector proteins, such as EGFR or RAS/MAPK reactivation, which can restore the proliferation and survival even when the original ALK fusion remains inhibited. The mechanism underlying the alectinib and lorlatinib-resistant cases is summarised in the earlier section (65). On-target resistance often involves point mutations within the ALK kinase domain, including the G1202R substitution described in the uterine IMT case, as discussed in Section 6.2, which hinders the action of first- and second-generation inhibitors. Crizotinib reduces target occupancy and increases the dosage of the fusion protein in double-fusion events such as *PLEC-EML4-ALK*. A less characterised third category is the non-genetic adaptation, which includes epigenetic remodelling and phenotypic plasticity, which can reduce the dependence on the original oncogenic driver without identifying secondary mutation (67). Since these mechanisms can co-occur and evolve in the same patient, serial liquid biopsy-based monitoring rather than a single baseline genomic assessment is necessary to understand the resistance landscape.

### 8.2 Anti-inflammatory therapies: clinical experience and limitations

Before modern molecular therapies, glucocorticoids and NSAIDs were the mainstay of IMT treatment. In early paediatric cases, corticosteroids and COX-2 inhibitors occasionally produced partial or complete responses, likely through inflammatory suppression and modulation of cytokine signalling. However, these responses were inconsistent and short-lived, reflecting IMT’s dual nature as both an inflammatory mass and a neoplasm. While corticosteroids and NSAIDs can relieve symptoms and occasionally stabilise imaging findings, their long-term efficacy in controlling tumour growth is limited (82). Kinase inhibitors, particularly crizotinib, ceritinib, and entrectinib, in ALK- or ROS1-rearranged IMTs have demonstrated higher response rates and delayed disease progression. Inflammation-targeted therapies are therefore best utilised as a complementary strategy rather than a standalone treatment approach (48).

### 8.3 Integration of cell cycle targeted drugs

Molecular approaches have become central to IMT management, particularly given the prevalence of actionable kinase fusions like ALK and ROS1. Targeted inhibitors such as crizotinib are now standard for unresectable or metastatic fusion-positive IMT, often producing rapid and durable responses. However, treatment options remain limited for fusion-negative cases, incomplete responders, or those who progress on TKIs, making cell-cycle targeting a logical avenue for combination therapy. For fusion-positive IMT, targeted therapy such as crizotinib is the first-line approach. Upon progression, adding checkpoint inhibitors like Wee1 or CHK1 inhibitors alongside chemotherapy may be considered, particularly when genomic analysis reveals p53 pathway disruption (48). For fusion-negative or refractory IMT, molecular profiling should guide treatment selection. CDK4/6 inhibitors are appropriate when RB is intact with p16/CDKN2A loss, whereas Wee1/CHK1/ATR inhibitors may suit tumours that exhibit replication stress or G1 checkpoint dysfunction. Given that timing and sequencing significantly influence whether combinations are synergistic or antagonistic, these agents are best evaluated within clinical trials (83).

It should be emphasised that at present there is no clinical or preclinical data for IMT that directly supports the use of CDK4/6 or Wee1/CHK1/ATR inhibitors. CDK4/6 inhibitors, including palbociclib and ribociclib, have shown CDK4/6-dependent activity against sarcoma with the loss of Rb and CDKN2A, with well-differentiated and dedifferentiated liposarcoma (84, 85). In the same way, CHK1 and ATR inhibitors are also shown to have preclinical activity in fusion-driven paediatric sarcomas in which fusion oncoprotein itself generates replication stress that can be therapeutically exploited. Before considering these agents for IMT, preclinical work on patient-derived IMT models or organoids is required to confirm the relevant vulnerabilities, such as Rb status, CDKN2A loss or replication stress markers (86, 87).

Overall, integrating cell-cycle-targeted therapies offers a flexible strategy for broadening treatment options in IMT, helping transition its management from a fusion-driven approach to one that exploits a broader range of vulnerabilities in cell-cycle control.

## 9 Conclusion

Inflammatory myofibroblastic tumours are inflammatory spindle cell lesions with a low metastatic rate and a high risk of recurrence. The rarity of IMT, the lack of precise diagnostic criteria, non-specific symptoms, and its similarity to other lesions often pose challenges in its identification and treatment. A total surgical removal is still the gold standard of treatment when possible; nevertheless, ALK targeted therapy is extremely important in ALK-positive inflammatory myofibroblastic tumours. Future research on immunotherapy, radiotherapy, and other molecular targets, such as ROS1, is necessary to lift the shroud of mystery around IMT. Furthermore, the computational drug design process using modelling and docking software will help develop drugs targeting the latest ALK fusion genes.

Additionally, next-generation sequencing (NGS) is essential for improving our understanding of the mechanisms underlying resistance in ALK fusion-positive cancers and for elucidating the oncogenes at play. This would help develop inhibitors targeting specific molecular markers, leading to more successful therapies by enabling personalised treatments based on the tumour’s genetic profile.

Altogether, the review here points to a central theme: the macrophage-rich, cytokine-driven inflammatory microenvironment of IMT is not merely a bystander but is fused with the oncogenic ALK/ROS1/NTRK signalling, with shared nodes such as the NF-κB and IL-6/STAT3 axes. This fusion kinase activity creates an inflammatory milieu, which in turn supports PD-L1-mediated immune evasion, macrophage recruitment, and myofibroblast proliferation, thereby creating an inflammation-oncogenic loop that drives tumour persistence and the emergence of resistance to targeted therapy. Understanding this loop reframes IMT as a disease where inflammatory and oncogenic signalling are mutually dependent, with direct action of combining ALK-directed therapy that targets the macrophage/cytokine axis. Future research priorities should focus on prospective-based studies that generate IMT-specific efficacy data for ALK and ROS1 inhibitors, mechanistic and translational studies on TAM polarisation, PD-L1 expression, and IL-6/STAT3 signalling to treatment response and resistance, and, finally, on preclinical development of patient-derived IMT models that can be evaluated for cell-cycle-targeted agents.
